# Engagement of African American Women With Fitness Trackers and Mobile Technology for Shared Physical Activity Goals: Mixed Methods Study

**DOI:** 10.2196/68006

**Published:** 2025-12-30

**Authors:** Allison Sweeney, Timothy Simmons, Anika Nair, Lindsay Decker

**Affiliations:** 1 Department of Biobehavioral Health and Nursing Science, College of Nursing, University of South Carolina, 1601 Greene Street, Columbia, SC, 29208, United States, 1 803-576-7891; 2 Department of Psychology, University of Maryland, College Park, Maryland, United States

**Keywords:** African American women, physical activity, social support, mHealth, mobile health, African American, fitness tracker, activity tracker, multimethods approach, Fitbit, mobile app, physical activity intervention

## Abstract

**Background:**

While there is growing evidence demonstrating the usefulness of integrating social features within mobile health approaches, little research has explored how African American women use mobile platforms to facilitate physical activity within the context of a group-based physical activity intervention.

**Objective:**

The primary aim of this study was to qualitatively describe how African American women used private group messaging boards on the Fitbit mobile app (eg, the type of social and motivational strategies) during a 10-week group-based physical activity intervention. The secondary aim of this study was to quantitatively test whether greater engagement on the Fitbit mobile app (number of posts per week) was associated with greater daily physical activity (ie, steps and total minutes of physical activity) across 10 weeks.

**Methods:**

Data were collected from 54 African American women who participated in the Together Everyone Achieves More Physical Activity trial (mean age 51.57, SD 13.89 y). Participants completed weekly in-person group sessions, set group-based weekly physical activity goals, and used the Fitbit mobile app for ongoing group communication and support, including posting in a private group. This study used a QUAN-qual mixed methods design to describe how participants used the private group messaging boards on the Fitbit mobile app and to evaluate whether engagement on the Fitbit app was associated with greater physical activity (ie, steps and total minutes of physical activity) across the 10-week intervention.

**Results:**

The mean number of posts per week ranged from 1.79 (SD 2.95) in week 1 to 1.11 (SD 2.49) in week 10, with a maximum of 5.06 (SD 7.62) posts in week 5. A thematic content analysis revealed that the private groups reflected numerous topics, including motivational strategies, cognitive strategies, group strategies, informal positive communication, and sharing pictures. The quantitative analyses revealed that participants who posted at least once per week engaged in more steps (*P*=.006) and total minutes of physical activity (*P*=.02).

**Conclusions:**

Participants engaged in ongoing social support, positive communication, and vicarious learning through the Fitbit app, suggesting several important directions for future research, including potential interpersonal mechanisms and best practices for enhancing social support and physical activity among African American women.

## Introduction

Although there have been notable improvements in cardiovascular disease mortality in recent decades, relative to other demographic groups, African American women are burdened with significantly higher rates of cardiovascular disease and are at greater risk for premature death from cardiovascular disease from earlier ages [1,2]. Participating in regular physical activity (PA) reduces risks associated with type 2 diabetes, cardiovascular disease, and cancer and reduces symptoms of anxiety and depression [3-5]. Numerous studies have highlighted the importance of social environmental factors for promoting PA, with African American women reporting a desire for various interpersonal forms of support, including being physically active with family or friends, receiving tangible support with caregiver or household responsibilities, having positive African American female role models, and greater exposure to other physically active African American women [6-10].

Efforts to promote social support for PA in past interventions for African American women have often drawn from in-person group-based approaches [11-14], which offer rich opportunities for creating a sense of belongingness and group cohesion [10,15]. Such programs have used a variety of interpersonal strategies, including group discussions of social support (eg, how to get support from family and friends and types of social support) [11,12,14] and partner-based approaches (eg, identifying a partner to be active with) [13,16]. Increasingly, there is growing evidence that smartphone apps, fitness trackers, and participation in mobile health (mHealth) interventions are feasible and acceptable among African American women [17,18], which has led to additional efforts to integrate mHealth platforms as an additional approach for enhancing social support for PA among African American women [16,19-21].

Several studies have provided preliminary support for the importance of integrating social features within mHealth interventions targeting PA among African American women (eg, group communication and sharing PA progress). For example, one study included a small group intervention where African American women received a mobile app that allowed them to see Fitbit data from 3 other participants and use instant message chatting [20]. Participants in the small group intervention were more likely to meet a goal prescribed by the research team (ie, at least 90 min of light activity per day for 3 mo) compared to those who were assigned to use a mobile app without the group-based features. Another study with African American women evaluated a culturally tailored mobile app intervention, which integrated individuals’ Fitbit data and included social support features, such as multimedia modules, text messages from the research team, and participant discussion forums [21]. Compared to those who received a wellness-focused mobile app, those with the intervention app demonstrated positive increases in self-reported PA.

While there is growing evidence highlighting the overall usefulness of integrating social features within mHealth approaches, little research has explored how African American women use mobile platforms to facilitate PA within the context of a group-based PA intervention. There are a variety of ways in which individuals could use the social features of mobile technology to facilitate PA, such as different types of social support (eg, words of encouragement and sharing information or resources), social facilitation (ie, observing others’ PA levels and activities), and friendly competition (eg, participating in step “challenges” and leaderboard rankings). Importantly, previous studies have found that simply providing African American women with a fitness tracker and encouraging them to use an affiliated smartphone app within their own social network may not be sufficient for increasing PA [22]. Instead, it may be important to provide additional structure to encourage interactions between peers who share similar goals related to PA.

This study uses secondary data from the ongoing Together Everyone Achieves More Physical Activity (TEAM-PA) randomized controlled trial, a group-based social affiliation intervention for increasing total daily PA among African American women using a randomized group cohort design [19]. A unique aspect of the TEAM-PA intervention is that it combines weekly in-person group sessions, group-based goal setting, fitness trackers (ie, Fitbits), and the Fitbit mobile app to facilitate ongoing communication and support among group members. The TEAM-PA intervention draws from group dynamics theory [23,24] and related studies [25-27], which propose that group-based goal setting is an effective approach for enhancing group cohesion, social support, effort, and motivation. While previous interventions have typically emphasized either in-person group sessions [11-14] or a mobile platform for delivering an intervention [20,21], the TEAM-PA intervention capitalizes on both through in-person group sessions (with opportunities to build a positive group climate and social support through group discussions, team-based PA games, and group-based goal setting) and encouraging participants to use a private group on the Fitbit mobile app throughout the week to facilitate group support and communication.

This mixed methods study aimed to expand upon previous research by providing an in-depth evaluation of how African American women use mobile technology to pursue shared group-based PA goals and the relationship between mobile app engagement and PA, which has not been directly evaluated in previous research. Importantly, identifying prominent strategies may help to inform best practices (eg, features to include in future mHealth approaches) and expand our understanding of potential modifiable interpersonal mechanisms for future interventions (eg, social support, social facilitation, and friendly competition). The primary aim of this study was to qualitatively describe how African American women used private group messaging boards on the Fitbit mobile app (eg, the type of social and motivational strategies) during the 10-week group-based TEAM-PA intervention. The secondary aim of this study was to quantitatively test whether greater engagement on the Fitbit mobile app (number of posts per week) was associated with greater daily PA (ie, steps and minutes of total physical activity [TPA]) throughout the 10-week intervention period.

## Methods

### Study Design

Data were collected for secondary analysis from the TEAM-PA trial [19]. The TEAM-PA study is an ongoing randomized controlled trial testing the efficacy of a novel group-based intervention versus a standard group-delivered PA comparison program for increasing total daily PA among insufficiently active African American women [19,28]. The TEAM-PA trial uses a group cohort design, with each cohort consisting of 3 to 4 groups, with 10 to 15 participants per group. Both the intervention and comparison programs involve weekly 2-hour group sessions for 10 weeks implemented at separate community sites and are co-led by 2 trained African American female facilitators. As the comparison group did not emphasize the use of the Fitbit mobile app, this study focuses on participants (N=54) randomized to the TEAM-PA intervention in cohorts 1 and 2, which included a total of 4 intervention groups, implemented between September 2022 and May 2023. This study uses a QUAN-qual mixed methods design guided by Morse and Niehaus [29] to describe how participants used the private group messaging boards on the Fitbit app and to evaluate if engagement with the Fitbit mobile app (number of posts) is associated with greater PA (ie, steps and TPA) across the 10-week intervention period.

The TEAM-PA intervention combines elements from social cognitive theory [30], self-determination theory [31], group dynamics theory [23,24], and a focus on collectivism [32] to integrate different components of social affiliation (eg, relatedness, reciprocal support, group cohesion, and collective efficacy). Drawing from this multitheoretical framework, the TEAM-PA intervention targets (1) collaborative skills and support, including the use of group-based behavioral skills (shared goal setting and problem-solving) and peer-to-peer positive communication; (2) friendly intragroup competition (including opportunities for peer-to-peer challenges, social facilitation, and optimal challenge); and (3) a collectivism focus, including emphasizing the group’s PA progress and the discussion of relevant cultural topics.

### Participants

Participants were recruited in collaboration with local community partners and organizations, who assisted with distributing flyers and invitations to attend local community events (eg, health fairs, family nights, and church events). Eligible participants met the following criteria: (1) age >17 years, (2) self-identifying as an African American or Black female, and (3) engaging in <60 minutes of self-reported moderate to vigorous PA per week for the last 3 months. Exclusion criteria included (1) having a cardiovascular or orthopedic condition that would limit PA, (2) inability to walk without a walker or cane, (3) pregnancy, or (4) uncontrolled blood pressure (systolic >180 mm Hg or diastolic >110 mm Hg).

### Procedures

The weekly in-person group sessions follow a standard structure, including a health curriculum, intragroup competitive PA sessions, and group-based behavioral skills. The health curriculum included guided group discussions designed to target behavioral skills for promoting PA (eg, self-monitoring and getting social support), was culturally adapted to be relevant for African American women, and integrated topics related to collectivism. Participants completed 30 minutes of PA, including a warm-up, a competitive group activity (eg, relay-based games, calisthenics challenges, or team-based chair exercise challenges), and a cooldown, which was implemented by the trained facilitators. Finally, each week participants collaboratively decided upon a shared group-based PA goal (eg, walking at least 5000 steps, 3 d per week). Participants were provided with a Fitbit (Inspire) to track their PA and received instructions for using the Fitbit mobile app. Fitabase (Small Steps Labs LLC) was used to compile participants’ weekly Fitbit data and provide feedback about the group’s overall performance. Facilitators used open-ended prompts to facilitate group-based behavioral skills training, including identifying strengths, areas for improvement, sources of support, anticipated barriers, and brainstorming solutions.

Outside of the group sessions, participants used the Fitbit mobile app to facilitate meeting the weekly group-based PA goal. Participants were linked as “friends” to other group members on the app, and a private group was set up by the research team to facilitate communication among group members. A voluntary “check-in captain” was selected by the group each week, who was responsible for creating at least one weekly post for the group. Participants were encouraged to use the app to encourage each other to meet the weekly group-based PA goal and communicate outside of the in-person session, including posting messages, updates, and photos. The group facilitators had access to the private group and were responsible for posting the weekly group goal to the private group and checking in mid-week to reinforce participants’ posting and sharing. The Fitbit mobile app also included a group leaderboard, which automatically ranked a user’s total weekly steps in reference to their “friends” and allowed group members to see one another’s steps.

### Measures

#### Physical Activity (Steps and TPA)

Fitabase was used to compile daily steps and TPA (ie, light, moderate, and vigorous) during the 10-week intervention period [33]. Daily values were summed across intensities and averaged at the week level.

#### Engagement on the Fitbit Mobile App

The total number of posts to the private groups per week was calculated to evaluate engagement on the Fitbit mobile app.

### Data Analysis

#### Qualitative Analysis

A total of 752 posts from the private groups were extracted from the Fitbit mobile app for a thematic content analysis [34], including original posts, comments, and pictures. All items were deidentified using a unique participant ID number before qualitative analysis. Posts made by facilitators were not included in the analysis. Guided by the theoretical framework for the TEAM-PA trial, a preliminary coding book was developed using a deductive approach. During a training period, a subset of the items was reviewed using an inductive approach to identify additional codes [34]. The final coding book was reviewed by all co-authors to condense codes. Two evaluators (AN and TS) coded each item. Interrater reliability was high (*r*=0.80), and any coding disagreements were settled through discussion until 100% agreement was reached. The qualitative software NVivo was used to perform a content analysis and organize the results into primary and secondary themes, which are consistent with previous qualitative studies using a hybrid inductive and deductive approach [35,36].

#### Quantitative Analysis

Following the qualitative analysis, quantitative analyses were conducted to evaluate whether weekly engagement on the Fitbit mobile app (number of posts) was associated with weekly PA across the 10-week intervention period. A valid day of Fitbit wear was defined as at least 1000 steps per day, which is consistent with previous studies [16]. Participants had an average of 6.73 days (SD 0.59) of valid Fitbit wear per week. Before analysis, outliers were identified and recoded to reflect a maximum of 3 times the IQR using a Winsorizing approach [37]. This resulted in the recoding of ≤3% of weekly values for steps and TPA. The number of posts was positively skewed. To correct for this, this variable was recoded as a dichotomous variable, such that 0=no posts per week and 1=at least one post per week.

Mixed models were used to examine the associations between the number of posts per week and average weekly PA (steps and minutes of TPA). The model included a fixed effect for time, which represented the week of the program (ranging from 1 to 10). Restricted maximum likelihood was used for model estimation using the lmerTest R package [38]. To account for the nesting of participants within intervention groups, all models included a random intercept for intervention group and individuals within groups. Age was mean-centered, and all other covariates were coded as dichotomous variables to aid in interpretability, including children living at home (1=at least one child at home and 0=no children at home) and education (1=4-y college education or greater and 0=no college education).

### Ethical Considerations

All participants signed a University of South Carolina Institutional Review Board–approved informed consent before participation (Pro00113152), and the TEAM-PA trial was registered with ClinicalTrials.gov (NCT05519696) in August 2022 before participant enrollment. Participants completed informed consent and had the ability to discontinue participation at any time. All participant data were deidentified before analysis. As part of the larger TEAM-PA trial, participants were financially compensated with US $25 for the baseline, US $50 for the postintervention, and US $75 for the 6-month follow-up assessments.

## Results

### Participant Characteristics and Descriptives

Participants (N=54) were aged between 27 and 76 years, with an average age of 53.48 (SD 14.49) years. Approximately 55% (30/54) were married, and 32% (17/54) had at least one child living at home. The median annual household income was US $40,000 to US $54,999, and 61.1% (33/54) of participants had a college degree or greater. Approximately 69% (37/54) of participants were working, with 31 individuals indicating they worked full time. At baseline, the average BMI was 34.84 (SD 8.05) kg/m^2^, with 70.3% (38/54) of participants having a BMI in the obese range (≥30 kg/m^2^). During the 10-week intervention period, the mean number of posts per week ranged from 1.79 (SD 2.95) in week 1 to 1.11 (SD 2.49) in week 10, with a maximum of 5.06 (SD 7.62) posts in week 5. Approximately 63% (34/54) of participants averaged at least one post per week. Three participants never posted in the private group.

### Qualitative Results

Table 1 provides a summary of themes, including definitions, the number of sources (total number of participants whose posts reflected that theme), and the frequency of themes (total instances across all participants). The qualitative analysis resulted in the following primary themes: motivational strategies, cognitive strategies, group strategies, informal positive communication, and sharing pictures. Each primary theme included several secondary themes, as described below.

**Table 1.** Summary of the qualitative themes (N=54).

Primary themes and secondary themes		Definition	Sources	Frequency
Motivational strategies				
Words of encouragement—individual		Offering words of encouragement aimed at an individual, such as mentioning their name or addressing an individual in the comments	35	222
Sources of motivation		Discussion of broader reasons or sources of motivation for being physically active and values related to physical activity (eg, family and health)	19	47
Cognitive strategies				
Sharing ideas about ways to be active		Sharing ways to be physically active (eg, activities, places to be active, and home workouts)	37	163
Goals or effort or progress		Discussion of efforts to achieving the team goal, such as whether they met the team goal or progress for the day	36	193
Barriers or challenges to physical activity		Discussion of barriers or challenges for engaging in physical activity and strategies for overcoming barriers	5	5
Benefits of physical activity		Discussion of the perceived benefits of physical activity (eg, physical, emotional, and mental)	3	4
Group strategies				
Words of encouragement—group		Offering words of encouragement aimed at the whole intervention group	35	135
Leaderboard or competition or challenge		Discussion of the group leaderboard or friendly competitive banter about total steps and ranking on the leaderboard	16	26
Plans to meet up		Discussion of plans to meet up outside of the group sessions (eg, social events and group walks)	10	32
Informal positive communication				
Checking in or greetings		Brief message to check in or say hi to the group (without other content related to physical activity or goals)	30	84
Thanks or appreciation		Thanking another participant or expressing appreciation for their words, actions, posts, or comments	27	80
Religion or spirituality		Referring to religion or spirituality through prayers or quotes (eg, for motivation, support, and advice)	23	49
Concern or sympathy		Expressing concern or sympathy for situations or individuals (eg, illness and family challenges)	17	23
General life updates		Discussion of friend, family, or life updates outside of the intervention group (not related to physical activity or goals)	17	55
Healthy meals or recipes		Discussion of healthy meals or recipes	17	50
Humor or joking		Making jokes or using humor in posts	14	32
Holidays or celebrations		Discussion of holidays or celebrations	8	13
Sharing pictures				
Using the Fitbit		Pictures related to using the Fitbit or the Fitbit app (eg, badges, step count, workouts, and tracking)	29	123
Picture of participant(s)—check in		Pictures taken of 1 or more participants to provide a general update to the group (eg, meet-ups, selfies, images of nature, and activities with family)	28	110
Graphic with motivational message		Pictures sent to motivate and spread positivity to other participants (eg, memes and GIFs)	19	91
Places to be active		Pictures showcasing where participants are being physically active (eg, neighborhood, parks, gym, and home)	16	64
Engaging in physical activity		Pictures showcasing different types of physical activity (eg, walking and physical activity equipment)	11	23
Healthy food or meals		Pictures showcasing healthy meals or food	10	9
Strategies for being physically active		Pictures showcasing strategies for being physically active were discussed in the group sessions (eg, how to get more steps or daily physical activity)	6	8

### Motivational Strategies

More than half of the participants posted words of encouragement aimed at an individual within the group, such as “Let’s get moving” in response to another participant’s post. Other examples included “You look happy. Keep moving,” “Go girl you are doing great,” and “Beautiful! Keep stepping!” Participants also posted about their various sources of motivation for PA. One participant described the role of family as a motivator, “My son is sooo proud of me for taking the 10 weeks class and moving forward with you and our team throughout the months ahead.” Another participant noted that music was an important source of motivation for her, “Starting today with rock steady Aretha Franklin and dancing with Michael Jackson.” Other sources of motivation included seeing what others in the group were doing, such as “The fact that I was even close to your steps numbers really motivated me this week,” and wanting to be a role model for oneself or others, “Now, how many steps will you take, knowing that someone is following you to victory? Lead by example.”

### Cognitive Strategies

More than half of the participants generated posts in which they shared ideas about ways to be active, including sharing where they were being active, such as “Getting a few steps in at the SC State Fair” or “Getting in some steps at the Pecan Festival with my son.” Participants also sometimes shared who they were being active with, such as “Hi guys I’m so happy my parents are here from Louisiana and my dad and I are getting in some steps.” Another prominent cognitive strategy involved discussing goals, effort, or progress toward meeting the group goal. For example, 1 participant posted about her determination to reach the group step goal, “It’s a little cold out here today, but the coldness is not going to stop me from getting my laps in. I’m taking Mrs. [name omitted] advice & try to reach my goal before the sun goes down.” Another participant shared her plans to catch up and meet the group goal for the day, “I am presently many many many steps behind. I am going to dig deep within me to get charged and to get energized for today’s steps.” Additional cognitive strategies included discussing barriers and challenges to PA (eg, “Yesterday was a struggle for me. It was one of those ‘I don’t feel like working’ kind of days. But I pushed through to this awesome new day and I’m optimistic about meeting the goal for today”) and the benefits of PA (eg, “definitely a good stress reliever to run today”).

### Group Strategies

In addition to responding to individual posts or users, participants also engaged in several group-based strategies. For example, more than half of the participants expressed words of encouragement geared toward the whole group, such as “Hi ladies let’s get some extra steps in today I know we got this you can do it!” or “Good morning and happy Friday ladies let’s make today count you can do it.” Another group strategy involved discussion of the group leaderboard or competition or challenge, such as, “Today, I want to do better, so early in the morning, I did the [group] challenge. Almost 2000 steps completed! Have a great day!” Another group strategy involved discussion of plans to meet up in person to be active together, such as, “Hey guys ‘relay for life’ is tomorrow Friday at 5PM −1030pm at [location omitted] some of the ladies will be meeting there to walk.”

### Informal Positive Communication

Participants also generated numerous posts that served as informal positive communication among group members. The 3 most frequent forms of communication included checking in or greetings (eg, “Everyone missed you ladies yesterday, but unexpected work meetings are always great....can’t wait for next week”), expressions of thanks or appreciation (“Good morning I miss y’all too it’s not often that you meet a group of ladies and bond so easily with them. Enjoy your weekend ladies”), and references to religion or spirituality (eg, “Lifting you up in prayers. Take care of yourself!”). Other types of informal communication included expressions of concern or sympathy (eg, “So sorry to hear that! Hope you feel better real soon! We’ll send a prayer for you and we will also miss seeing you tomorrow”), sharing about general life updates (eg, “[visiting] Stone Mountain Georgia with my family”), sharing recipes or ideas for healthy eating (eg, “I cooked Alfredo with grilled chicken, shrimp, and broccoli for dinner but instead of regular white noodles I used whole wheat noodles and the kids and hubby tore it up so I’ll be switching out my white noodles for wheat”), humor or joking (eg, “Me! When I realized that my Fitbit was dead during the second lap. Lol Don’t worry I kept walking!”), and sharing about holidays or celebrations (eg, “Happy Thanksgiving blessings to you and your family”).

### Sharing Pictures

Participants’ posts were often accompanied by pictures, including pictures taken by participants, screenshots, and other images, which were coded separately from text-based comments. The most prominent category of pictures was images related to using the Fitbit, including documenting their physical activities, accomplishment “badges,” workouts, the leaderboard, or use of other Fitbit content (eg, meditation, heart rate, and sleep). Participants also posted numerous images to check in with the group, such as documenting their daily activities, meet-ups with other participants, selfies, images of nature, and activities with family or friends. Several participants shared screenshots of graphics or memes with motivational messages. Participants also frequently posted images of places where they were being active, including places in their neighborhood, parks, and recreation centers. Other categories included images of participants engaging in PA (eg, walking and PA equipment), images of healthy food or meals, and documenting strategies for being physically active discussed in the group sessions (eg, parking farther away to get more steps and using the stairs at work).

### Quantitative Results

As presented in Table S1 in Multimedia Appendix 1, a mixed model was used to examine the association between weekly posting in the private group and average weekly steps per week across the 10-week intervention period. There was a significant main effect of number of posts on steps (estimate 585.58, SE 212.04; *P*=.006), such that individuals who posted at least once per week engaged in greater weekly steps. Similarly, as presented in Table S2 in Multimedia Appendix 1, the same modeling approach was used to examine the association between weekly posting in the private group and TPA. Again, there was a significant main effect of posts on TPA (estimate 13.94, SE 5.93; *P*=.02) such that individuals who posted at least once per week engaged in greater weekly TPA. We also investigated whether the effect of posts was moderated by time but did not observe any 2-way interactions, suggesting that the effects of engagement with the Fitbit app on steps and TPA were relatively stable across the 10-week intervention period.

## Discussion

### Principal Findings

This study evaluated the types of social and motivational strategies used by African American women on a mobile platform as part of a group-based PA intervention and whether weekly posting in the mobile app was associated with PA across the 10-week intervention period. Overall, the qualitative and quantitative components of this study provided complementary perspectives on the role of engagement with private group messaging boards as part of a group-based PA intervention for African American women. The qualitative results indicated that communication in the private groups reflected numerous topics, including motivational strategies, cognitive strategies, group strategies, informal positive communication, and sharing pictures. Some of the most prominent secondary themes included words of encouragement (individual and group); sharing ideas about ways to be active; discussion of goals, effort, or progress; and sharing pictures (eg, using the Fitbit, picture of participants—check-in, and graphics with motivational messages). Furthermore, the quantitative analyses revealed that weekly posting in the private group was associated with greater PA, such that participants who posted at least once per week in the private group engaged in greater steps and TPA across the 10-week intervention period.

The qualitative results suggest that participants were engaged in different forms of social support within the private groups, including emotional social support (eg, words of encouragement), informational support (eg, sharing ideas and images of ways and places to be active), and other forms of support (eg, making plans to be active together). According to social cognitive theory [30], social support is important for promoting self-efficacy, and both vicarious experiences (eg, observing similar others be successful and role modeling) and positive verbal persuasion (eg, words of encouragement about an individual’s capability) are essential social support factors [39]. Other secondary themes, such as discussion of goals, progress, or efforts and sharing pictures related to the Fitbit (eg, workouts, badges, and places to be active), may reflect role modeling and vicarious learning processes. Furthermore, self-determination theory [24] proposes that relatedness (eg, feeling valued or included by close others) is important for building autonomous motivation, which is considered a key predictor of long-term PA [39]. The informal positive communication observed in the private groups (eg, expressions of concern or sympathy, thanks or appreciation, and checking in or greetings) may also reflect the development of camaraderie and relatedness among group members. Taken together, these results suggest that future interventions may benefit from using mobile platforms to target different theory-based interpersonal mechanisms, including social support (emotional and informational), vicarious learning processes (eg, encouraging participants to share PA progress and pictures), and relatedness (eg, encouraging informal positive communication that facilitates a sense of belongingness).

By demonstrating that engagement with the private group messaging boards was associated with greater PA, this study adds to growing evidence that combining fitness trackers with a mobile group platform with interpersonal features is a promising approach for increasing PA among African American women [16,20,21]. However, it is important to note that sustaining engagement with interventions delivered through mobile apps remains an ongoing challenge, with some studies finding notable variability across participant groups [20,21]. Importantly, unlike these previous studies, which were delivered entirely through a mobile app [20,21], the TEAM-PA intervention also included an in-person component with weekly group-based sessions, which may have allowed for additional opportunities to build rapport and, in turn, impacted engagement with the Fitbit mobile app. Furthermore, the inclusion of group-based goals, which have been found in previous studies to enhance group cohesion [23-27], may have also increased participants’ engagement with the Fitbit mobile app (eg, interest in seeing the group’s progress toward the weekly goal). Group-based goals and the emphasis on group performance may also be a more culturally congruent approach for African American women (as opposed to traditional individual-based goal-setting), as collectivism and group connectedness are often viewed as core cultural values among African American communities [32,40]. Thus, future interventions for African American women may benefit from integrating mHealth approaches that facilitate group communication and support with some in-person interactions and capitalizing on group goal setting to further strengthen social support and engagement.

### Strengths and Limitations

There are several strengths of this study, including the use of both qualitative and quantitative methods, objectively measured PA via Fitbits and a focus on an underrepresented population. While previous mHealth PA interventions for African American women have included features that allow for communication across participants (eg, message boards, private chatting), this study suggests that it may be important to use prompts or activities that specifically target different types of social support (emotional and informational), vicarious learning processes, discussion of goals and progress, and capitalize on group performance (eg, shared goal setting). Although previous studies have evaluated use of public social media platforms geared toward engaging African American women in PA (eg, Black Girls Run Facebook page [41]), to our knowledge, this is the first study to leverage both qualitative and quantitative approaches to evaluate engagement with a mobile platform within the context of a group-based intervention. The qualitative approach allowed for an in-depth assessment of how participants engaged with the private messaging boards, whereas the quantitative analysis demonstrated a significant association between engagement and PA behavior. One limitation is the relatively small sample size, which limits generalizability of the quantitative results, and further research is needed to evaluate generalizability to other demographic groups. Furthermore, we cannot infer the direction of the relationship between engagement with the Fitbit mobile app and PA. That is, it may be that participants who were more successful with reaching the weekly PA goals were more inclined to engage with their group members on the app or that engagement on the Fitbit app facilitated greater PA.

### Conclusions

This study demonstrated that as part of a group-based intervention, African American women were engaged with the private group messaging boards on the Fitbit mobile app to facilitate different types of social support, positive group communication, and reciprocal vicarious learning processes. While previous studies have tended to focus on Fitbits as a means for self-monitoring and PA adherence [42], this study highlights the usefulness of the social and communication-based features. Importantly, engagement with the private groups on the Fitbit mobile app was associated with greater weekly steps and TPA, suggesting that future interventions with fitness trackers may benefit from including and evaluating opportunities for participants to engage with other participants (eg, private groups) to more clearly determine the best practices for building social support and, in turn, promoting PA and intervention engagement and adherence.

## Supplementary material

10.2196/68006 Multimedia Appendix 1 Supplementary results tables.

## Abbreviations

PA: physical activity
TEAM-PA: Together Everyone Achieves More Physical Activity
TPA: total physical activity
