# Supplementary material for: Engagement of African American Women With Fitness Trackers and Mobile Technology for Shared Physical Activity Goals: Mixed Methods Study
Source: JMIR Mhealth Uhealth. 2025 Dec 30;13:e68006. doi: 10.2196/68006 (PMC12752912; doi:10.2196/68006)
Supplement: Multimedia Appendix 1 [file mhealth-v13-e68006-s001.docx]

**Multimedia Appendix**

Table S1. Mixed model predicting average daily steps per week

| *Predictors* | *Estimates* | *SE* | *95% CI* | *P* |
| --- | --- | --- | --- | --- |
| **Fixed Effects** |  |  |  |  |
| Intercept | 8616.56 | 1234.66 | 6191.09 to 11042.03 | <0.001 |
| Age | 38.66 | 31.7 | -23.61 to 100.93 | .223 |
| Children | 830.27 | 941.25 | -1018.80 to 2679.35 | .378 |
| Education | -1216.86 | 815.3 | -2818.49 to 384.78 | .136 |
| Week | 13.96 | 30.66 | -46.27 to 74.20 | .649 |
| Posts | 585.58 | 212.04 | 169.03 to 1002.13 | .006 |
| **Random Effects** | *Variance* | *SD* |  |  |
| Group \| ID | 7594697 | 2756 |  |  |
| Group | 3687359 | 1920 |  |  |
| Residual | 4036775 | 2009 |  |  |

Table S2. Mixed model predicting average daily minutes of total physical activity per week

| *Predictors* | *Estimates* | *SE* | *95% CI* | *P* |
| --- | --- | --- | --- | --- |
| **Fixed Effects** |  |  |  |  |
| Intercept | 303.88 | 30.82 | 243.34 to 364.42 | <0.001 |
| Age | 0.45 | 0.83 | -1.18 to 2.08 | .585 |
| Children | 13.1 | 24.63 | -35.28 to 61.49 | .595 |
| Education | -54.41 | 21.34 | -96.33 to -12.49 | .011 |
| Week | 1.63 | 0.86 | -0.06 to 3.32 | .058 |
| Posts | 13.94 | 5.93 | 2.29 to 25.58 | .019 |
| **Random Effects** | *Variance* | *SD* |  |  |
| Group \| ID | 5163 | 71.86 |  |  |
| Group | 2130 | 46.15 |  |  |
| Residual | 3164 | 56.25 |  |  |
